# Illuminating Artistic Processes toward Transdisciplinary Discourse

**DOI:** 10.3389/fnhum.2017.00522

**Published:** 2017-11-06

**Authors:** Ruslana Lichtzier, Philip Peters

**Affiliations:** ^1^Museum of Fine Arts, Houston, TX, United States; ^2^Independent Artist, Chicago, IL, United States

**Keywords:** aesthetics, neuroaesthetics, art, vision, creativity, beauty, transdisciplinary, contemporary art

What can we learn about aesthetics by studying the creative process of art-making? Much research has focused on the viewer's relationship with the art-object. Analyzing this experience provides a quantitative approach, but such an approach, as Chatterjee (2011) describes, runs the risk of “looking for the dropped coin under the lamp because that is where things are visible…” In this paper, we shift from the work of art as a site of inquiry, and instead, investigate and demystify some of the processes, attitudes, and systems that lead up to the production of artwork.

Through structured conversations, we discussed the creative work processes of five American artists: Kendall Babl, Iris Bernblum, Ryan Coffey, Danny Giles, and Kelly Lloyd. At the time we conducted the conversations all of these artists were based in Chicago, IL, USA, but their backgrounds, age, gender, ethnicity, and artistic production varied. The conversations followed four areas of inquiry: Seeing, Thinking, Context, and Beauty. Upon reviewing the conversation transcripts, we looked for commonalities, and created the following meta categories based on the artists responses: *Artistic Processes, The Expanded Studio, Learning to See*, and *Emotional and Spiritual Comprehension of Art and the Human Experience*. In the following sections, we outline how each of these categories were utilized by the individual artists.

Similar types of conversation have been employed in the field of creativity research (Csikszentmihalyi, 1996; Mace, 1997; Mace and Ward, 2002; Rawlings and Nelson, 2007; Botella et al., 2013) to cite a few. Our work differs in a few important ways. This body of research, which has its roots in Graham Wallas' four stages of the creative process (Wallas, 1926), details a scientific inquiry into the artist's creative process as separate from research into aesthetics. We do not support the separation of creativity research from aesthetic research and believe that the combination of both fields can advance the study of creative practices within the aesthetic realm. Tinio (2013) similarly attempts to link the two fields through his mirror model which contends that the experience of *art-viewing* is mirrored and analysable in that of *art-making*: “Considering the aesthetic processing of an artwork in terms of the artistic processes that produced it allows for an account of the experience of art in its fullest manifestation…”

Further, while summarizing his seminal and expansive research initiative, Csikszentmihalyi (1996, p. 14) laments “More than half of the natural scientists…agreed to participate. Artists, writers, and musicians, on the other hand, tended to ignore our letters or declined—less than a third of those approached accepted.” Our engagement with this subject is as cultural producers working from the inside. At the time we began our investigation, we relied on our intimate familiarity with artistic processes to first solicit and then guide the conversations. As Chatterjee (2014) states, “[Neuroaesthetics] is still working out its research agenda, methods of investigation, and even which questions are worth pursuing.” By documenting, and providing elemental analysis of these types of conversations, we hope to help shape future neuroaesthetic research and open new territory in the field of aesthetics, offering an interdisciplinary approach to the study of artistic process that includes collaborations between neuroscientists, artists, curators, and art-writers.

## Artistic processes

The entrance to a productive mindset often begins before the physical entrance to the space of the studio. Driving, riding a bike, or using public transportation on the way to the studio can become a space of transition to a productive mindset. A different space of transition will occur in the studio, where the artists may just “do nothing” for a while, shifting to a mode of concentration. Other times, these artists will start by cleaning up the studio. This allows them to start moving things around, initiating a simple process of decision making, such as the possible reuse or rearrangement of specific materials for the production of new work. Then, they may pick up a random object, and begin manipulating it without putting too much thought or intention into it, which opens a space for improvisation. While preparing to make work, the artists tended to focus on things that proved to spark their mind and senses, such as intellectually stimulating conversations and reading. Babl and Giles describe this as producing “obsessive” or “circling” thought processes that may help to initiate the work.

While the artistic process itself is said to be “intuitive,” we did succeed in tracing eight common stages. The artists adapted these stages in different ways, though they tended to be circular, meaning one may expect to cycle through these stages repeatedly until the end product appears. Not all stages applied to all of the artists at all times.

The eight stages are:

1. **Technical preparation:** setting up the materials to be worked on. This stage varies in time and runs the risk of over-occupying the artist's mind.
2. **Multitasking:** setting up multiple simultaneous material processes; this enables a circular flow, during which the artist can move from one object to another, and back, without “overworking” each element.
3. **Limitations:** applying rules that allow limited decision making.
4. **Play:** actions that do not have specific objectives.
5. **Mental imaging:** having a concrete, though at times abstract, mental image of an unrealized work.
6. **Problem solving:** identifying, structuring, and solving problems. “*Problems*” are moments in the process that do not yet *work*. The definition of a “problem” will vary from one practice to another.
7. **What works and what doesn't:** Within the studio process, a lot of the outcome “doesn't work.” In other words, the process entails a continuous failure that artists learn to work against. A work that “doesn't work” can be treated as a problem, then, it initiates a new studio cycle. At other times, the artist drops it, then, it's time to start over.
8. **The breaking point:** the moment when things become messy, when “the brain shuts down.” Sometimes it is the point where things get realized. If not, it is the point from which things will start over.

## The expanded studio

“Studio” is not only a specific space for work, but an encapsulating concept that includes both different spaces and practices that provide productive outcomes. Productivity in this case goes beyond the realization of a specific work, and expands to an aware interaction with the world. Through what Giles calls a “collapse of […] categories” the space of the studio can expand into the home, the classroom, the car, and the street. Among productive practices, artists listed walking, waiting, driving, and talking with friends and family. This allows them, as Giles puts it, to “multitask” between the different spaces, and to set simultaneous “processes in motion,” that provide a continuous flow that they “can dip in and out of.” Mental notes, sketches, and photos become a way of extending interaction with the work in different spaces. Lloyd and Babl expressed this when they spoke about dispersing the studio. The expansion and collapse of categories blurs the division between work and rest, reducing the negative attributes connected with “work,” and opening a space for a productive potential in seemingly unexpected places.

## Learning to see

One of the most fundamental, though rarely discussed elements of an artistic practice is the continuous self-learning process regarding the ways that human sight operates. Artists must learn how and what do they and others see to be able to produce effective artworks. This perspective takes into consideration that sight is not only a natural capability but also a cultural construct. In our conversations, artists identified through self-observation, a tendency of moving through the world without paying attention to their surroundings, and a general “habit” of selective seeing and noticing. Further, they have identified both visual literacy and aesthetic judgment as factors in the selective seeing process, meaning a general tendency to ignore what is not readily identifiable or visually appealing.

While Lloyd emphasized the importance of asking questions regarding the process, other artists' coping strategies differed. By way of example, we have identified two conflicting positions: Coffey interrogates his sight by working against what he is attracted to, while Giles trusts his eye, fine tuning his understanding to what he is attracted to.

Due to these opposite positions, the two artists developed different strategies to achieve their goals. When Giles notices something in the world, he follows his “instinct” and begins to seek out more of the same by keeping an attentive awareness.

Coffey's strategy is to challenge the direct gaze. Both in his studio and outside of it, he practices his peripheral vision. This, he believes, not only allows him to escape the trap of “I am *this* that looks at *this*,” but also to tap into a pool of wider information that the brain constantly processes. This alternative focus allows him, in his view, to produce work that expands the human experience.

As we mentioned earlier, aesthetic judgment is a fundamental factor that artists are attentive to while studying the complexities of sight. They defined beauty as the thing you gravitate toward, the thing that seduces you, and holds your attention, while also producing pleasure. Beauty has this effect not only because of natural characteristics, but also due to visual literacy. Artist's taste change with time and evolve, and may even reach a point of linking beauty with the abject.

Artists pay attention to their own taste as a factor in their decision making processes. Some follow their aesthetic preferences while others resist it. Those who resist, treat beauty as a territory worth investigating and problematizing. This in return becomes one of the basic characteristics of their artistic practice.

## Emotional and spiritual comprehension of art and the human experience

In our conversation artists expressed a strong association between making art and the process of continually realizing one's own humanity. Artists spoke about “being a human” (Lloyd), “the human experience” (Coffey), “what we think we are” (Babl), and making art as “crucial to your existence” (Bernblum). They defined their practices as modes of engagement with the world. While Giles highlighted the power of being “mindful” while going through life, Lloyd described making art as, “a kind of living,” and Coffey thought of art as a space that asks “what it is to be human.”

The artists expressed a belief in “being a part” of the process of making art, as opposed to being its sole authors. They described themselves as “agents” or as “facilitators” who find and reveal a work that is already there. They have emphasized “trusting” their process to bring them to the work; when a work reveals itself, it is as if “everything made sense” though it “just happens” by itself. While speaking about these experiences, the artists struggled the most to articulate their ideas. This should serve as a reminder that our discussion operates within the boundaries of language, while the experience exceeds it. As the artists we spoke with, we must also remain attentive to this gap, and remember the thing that escapes language, and that language itself is a limited tool to outline the aesthetic and creative experiences.

Artists perceive art making as a process that touches and reflects upon all aspects of human experience. This, as we previously exhibited, is a continuous process, where there is no clear delineation between practice and non-practice; the endpoint of art is not specific but aims to touch on and affect human experience as a whole.

## In closing

As cultural producers, one of the challenges we undertook in writing this paper was to try and mediate between the artistic and scientific communities across the contemporary field of aesthetics. Based on our conversations, it was clear that aesthetic experiences are not limited to the studio or the gallery and should be investigated as such. As Mace and Ward (2002) also described, there are “stages” attributable to artistic/creative behavior that could become the targeted investigation of future research. Not only did our artists train their faculties of sight, but in their art, sight itself became the subject of rigorous interrogation. Similarly they regarded beauty as a problematized concept to be manipulated though not necessarily achieved. The art historical discourse tells us that aesthetics is evoked by beauty, but to the contemporary artist, beauty and aesthetics are not equated, while sophisticated aesthetics are still achieved. Artists have complicated linkages between producing art and the human experience; art and the self are intertwined.

By continuing to speak with artists, and analyzing their self-reflective account of creative processes, a new channel can be used to structure scientific inquiries that better investigates its own questions of when, where, and how aesthetic experiences are manifested within the creative process.

## Author contributions

The opinion article “Illuminating Artistic Processes” was co-designed and co-written by PP and RL. Through structured conversations, the authors discuss the creative processes of five American artists: Kendall Babl, Iris Bernblum, Ryan Coffey, Danny Giles, and Kelly Lloyd. The conversations followed four areas of inquiry: Seeing, Thinking, Context, and Beauty. The paper identifies commonalities, and divided the artists' responses into the following categories: Artistic Processes, The Expanded Studio, Learning to See, and Emotional and Spiritual Comprehension of Art and the Human Experience. By documenting, and providing elemental analysis of our conversations, we hope to help shape this future research and open new territory in the field of Neuroaesthetics, offering an interdisciplinary approach to the study of artistic process that includes collaborations with artists, curators, and art-writers.

### Conflict of interest statement

The authors declare that the research was conducted in the absence of any commercial or financial relationships that could be construed as a potential conflict of interest. As is common practice within the arts community, given the limited number of local participants qualified to take part in these conversations, this project by necessity draws from a group of friends, family, and colleagues.
